# Assessing tick attachments to humans with citizen science data: spatio-temporal mapping in Switzerland from 2015 to 2021 using spatialMaxent

**DOI:** 10.1186/s13071-024-06636-4

**Published:** 2025-01-23

**Authors:** Lisa Bald, Nils Ratnaweera, Tomislav Hengl, Patrick Laube, Jürg Grunder, Werner Tischhauser, Netra Bhandari, Dirk Zeuss

**Affiliations:** 1 Faculty of Geography, Environmental Informatics, University of Marburg, Deutschhausstraße 12, 35032 Marburg, Hessen Germany; 2 Institute of Natural Resource Sciences, Zurich University of Applied Sciences ZHAW, Grüentalstrasse 14, 8820 Wädenswil, Zürich Switzerland; 3 OpenGeoHub Foundation, Cardanuslaan 26, 6865HK Doorwerth, The Netherlands; 4 A&K Strategy Ltd., Smartphone application “Tick Prevention”, Chastelstrasse 14, 8732 Neuhaus, Zürich Switzerland

**Keywords:** Citizen science, Lyme disease, Spatio-temporal mapping, spatialMaxent, Species distribution modeling, Switzerland, Tick attachment to humans, Ticks, Tick-borne encephalitis

## Abstract

**Background:**

Ticks are the primary vectors of numerous zoonotic pathogens, transmitting more pathogens than any other blood-feeding arthropod. In the northern hemisphere, tick-borne disease cases in humans, such as Lyme borreliosis and tick-borne encephalitis, have risen in recent years, and are a significant burden on public healthcare systems. The spread of these diseases is further reinforced by climate change, which leads to expanding tick habitats. Switzerland is among the countries in which tick-borne diseases are a major public health concern, with increasing incidence rates reported in recent years.

**Methods:**

In response to these challenges, the “Tick Prevention” app was developed by the Zurich University of Applied Sciences and operated by A&K Strategy Ltd. in Switzerland. The app allows for the collection of large amounts of data on tick attachment to humans through a citizen science approach. In this study, citizen science data were utilized to map tick attachment to humans in Switzerland at a 100 m spatial resolution, on a monthly basis, for the years 2015 to 2021. The maps were created using a state-of-the-art modeling approach with the software extension spatialMaxent, which accounts for spatial autocorrelation when creating Maxent models.

**Results:**

Our results consist of 84 maps displaying the risk of tick attachments to humans in Switzerland, with the model showing good overall performance, with median $\hbox {AUC}_{\textrm{ROC}}$ values ranging from 0.82 in 2018 to 0.92 in 2017 and 2021 and convincing spatial distribution, verified by tick experts for Switzerland. Our study reveals that tick attachment to humans is particularly high at the edges of settlement areas, especially in sparsely built-up suburban regions with green spaces, while it is lower in densely urbanized areas. Additionally, forested areas near cities also show increased risk levels.

**Conclusions:**

This mapping aims to guide public health interventions to reduce human exposure to ticks and to inform the resource planning of healthcare facilities. Our findings suggest that citizen science data can be valuable for modeling and mapping tick attachment risk, indicating the potential of citizen science data for use in epidemiological surveillance and public healthcare planning.

**Graphical Abstract:**

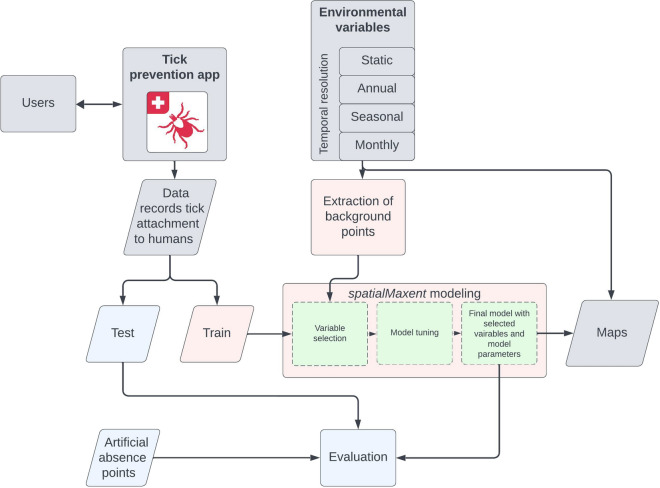

**Supplementary Information:**

The online version contains supplementary material available at 10.1186/s13071-024-06636-4.

## Background

Ticks (*Ixodes* spp.) serve as vectors for various zoonotic pathogens, including viruses, bacteria, and protozoa [1], and transmit the most pathogens among all blood-feeding arthropods [2]. Due to their generalist nature, ticks pose a potential threat by transmitting infectious agents to a vast array of hosts, exceeding 300 species, including mammals, birds, and reptiles [3].

In the temperate northern hemisphere, ticks play a considerable role as vectors for disease transmission to humans [4], with reported cases increasing in both the USA [5] and Europe [6] in recent years.

Of particular importance are the diseases tick-borne encephalitis (TBE) [7] and Lyme borreliosis [8], which is the most frequently transmitted tick-borne human disease in the world [9], with 153,120 reported cases in the year 2022 (Johns Hopkins Lyme and Tickborne Diseases Dashboard^1^). In 2022, the US Centers for Disease Control and Prevention reported 61,808 cases in the United States and estimated that 476,000 people may be diagnosed and treated for Lyme disease (Lyme Disease Surveillance Data, Centers for Disease Control and Prevention [CDC], USA, 2024^2^). However, ticks are also capable of transmitting numerous other diseases, including Alongshan virus [10], Rocky Mountain spotted fever [4], and Crimean–Congo hemorrhagic fever [11]. Tick-borne diseases are not only medically significant due to their high incidence rates, but they also pose a burden on the public healthcare systems [12, 13]. Furthermore, as tick-borne diseases keep spreading to new geographical areas and higher altitudes [14], the risk of infection is increasing for a larger human population, and climate change is also contributing to the further spread of tick-borne diseases [15].

Whether a person becomes infected with a tick-borne disease depends on the presence of ticks and whether they are carrying pathogens, but also to a large extent on human behavior. Contact with ticks in western countries occurs primarily during recreational activities such as hiking, as well as in green spaces within urban areas [10, 16, 17]. Data on direct tick attachment to humans can be challenging to acquire, and therefore, information on tick attachment to humans is typically extrapolated from disease cases and tick distribution patterns [16]. However, tick attachment to companion animals has already been mapped for Great Britain [18]. Many other previous studies have focused on mapping the habitat suitability of ticks [19], which can assist in understanding the drivers of their distribution. However, for healthcare planning purposes, it is valuable to also consider the spatio-temporal distribution of tick attachment to humans, as it has a significant impact on the strain placed on the healthcare system.

Switzerland is one of the countries where the incidence of tick-borne diseases has increased in recent years [20]. To address this, the Zurich University of Applied Sciences developed the “Tick Prevention” app, which uses a citizen science approach to collect data on tick attachment to humans [21]. Operated by A&K Strategy Ltd. [22], the app enables users to report tick bites and thereby provides crucial data for monitoring and understanding tick-borne diseases and spatio-temporal trends.

**Fig. 1:**
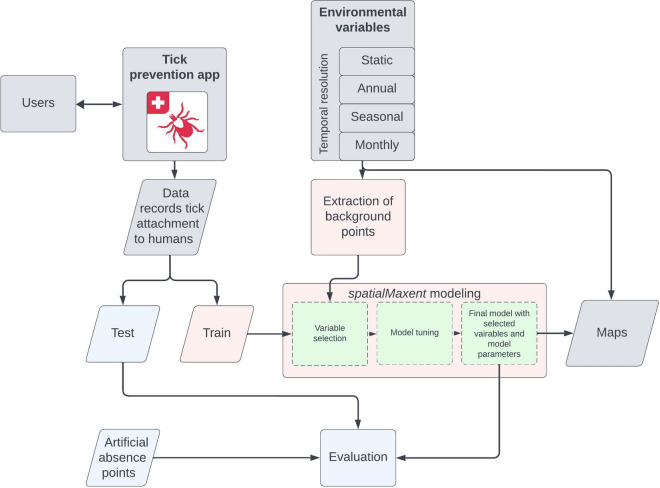
Workflow for mapping tick attachment to humans. Data on tick attachment to humans were collected through the Tick Prevention app. These data records served as training data for a model utilizing the software extension spatialMaxent, alongside background points derived from environmental variables. These environmental variables encompassed four temporal resolutions: static variables (e.g., digital height model), yearly data (e.g., population density), seasonal data (e.g., optical remote sensing data), and monthly data (e.g., weather data). During model creation, a variable selection process is initially conducted, where the most significant variables are automatically identified by the model. Subsequently, the other model parameters are tuned. The final model is trained with the best-performing parameters and variables. The performance of the model was assessed using artificial absence points derived from areas with minimal tick occurrence probability, such as lakes. Subsequently, 84 maps depicting tick attachment to humans from January 2015 to December 2021 were generated at a spatial resolution of 100 m

The data collected by the Tick Prevention app (Figs. 1, 3) can be upscaled with environmental variables to create wall-to-wall predictions using methods commonly applied in species distribution modeling (SDM), as both the data structure and modeling task are the same as those in SDM. SDM is widely used for mapping in various research areas [23]. However, Lee-Yaw et al. [24] pointed out that the majority of species distribution models perform poorly when tested against independent data, leaving modelers faced with many significant challenges. A possible explanation for the poor model performance could be the insufficient attention given to the spatial properties of the data, especially their spatial non-independence, during model training. Recent studies have emphasized the importance of accounting for the characteristics of spatial and temporal data when formulating validation and testing strategies in environmental modeling [25–28].

In order to overcome the above-mentioned challenges, this study addresses three major objectives: (1) to collect large-scale data on tick attachment to humans through a citizen science approach, (2) to apply a state-of-the-art modeling method that addresses current challenges in environmental modeling for enhanced reliability of the modeled results, and (3) to generate monthly maps of tick attachment to humans in Switzerland at a spatial resolution of 100 m from 2015 to 2021, providing information for both the population and policymakers.

## Methods

Data processing was executed with the use of the R packages sf (version 1.0.12; [29, 30]), raster (version 3.6.20; [31]), and terra (version 1.7.18; [32]). We used SDM to map the monthly tick attachment to humans (Fig. 1). The maps created in this study were modeled with spatialMaxent [33], a software extension for the SDM software Maxent [34]. All data processing was performed in R (version 4.3.1; [35]).

### Modeling approach

The data collected by the users of the Tick Prevention app (Fig. 1; Section "Tick reports") consisted of presence-only (PO) data. These data represent occurrence points of a species without providing information on where the species is absent. When additional data on species absences are collected, they are referred to as presence–absence (PA) data. Occurrence points of a species, or in this case of tick attachment to humans, can be utilized to generate area-wide predictions. To achieve this, PO or PA points are upscaled with comprehensive environmental variables to produce wall-to-wall predictions. Models that are created using PO points often require additional background points, also known as pseudo-absences [34, 36] (see Section "Background points/ pseudo absences"). These data are randomly or systematically sampled points throughout the whole study area, intended to cover the entire value space of the environmental variables used for modeling.

The data on tick attachment to humans used in this study share the same structure as species occurrence data typically used in SDM. Therefore, we apply software and methods commonly used in SDM. However, this does not imply that we are modeling species distribution; the term “SDM” is used in this study purely to describe the modeling approach. Since our data points represent tick attachment to humans, which inherently combines both tick dynamics and human exposure factors, it is not feasible to map these elements separately. Both factors must be simultaneously incorporated into the model, as the data points capture the interaction between them.

For the upscaling of PO data, various methods are employed in SDM to create area-wide maps. Some popular modeling methods, such as generalized linear models, generalized additive models, and support vector machines, although frequently used for other modeling tasks, perform less effectively for SDM [37, 38]. In contrast, methods like boosted regression trees, ensemble models, or Maxent have demonstrated a more favorable performance [37, 38]. For example, the software Maxent is a standalone Java software for SDM that uses a maximum entropy approach to create the species distribution models, and is very popular due to its good performance [37, 38] and user-friendliness [39]. In a review by Guillera-Arroita et al. [40] it was used in 41% of the reviewed SDM studies. Furthermore, it was specifically designed to create species distribution models with PO data [36].

As previously noted, poor SDM performance [24] may result from neglecting spatial data properties during model training, validation, and testing [25–28]. In particular, the use of random cross-validation in which test data are separated randomly from training data tends to result in an overestimation of model predictive power [26]. Furthermore, model tuning is also heavily influenced by the chosen cross-validation strategy [27, 28, 33]. The same applies to the selection of variables. It has been shown that automated variable selection yields superior outcomes compared to modeling approaches without such functionalities [27, 41]. If variable selection is conducted by an automated algorithm, it means that the modeler inputs all relevant variables into the model. The algorithm then tests which variables contribute to an improvement in model performance and which do not. Only the variables that enhance model performance are retained, while all others are excluded from the final model.

Therefore, we required a modeling method that (1) can handle PO data, (2) offers good performance compared to other methods, and (3) provides the capabilities for spatio-temporal cross-validation, automated tuning, and automated variable selection. For these reasons, we used the software extension spatialMaxent version 1.0.0 [33] for modeling and mapping of tick attachment to humans. spatialMaxent serves as an extension for Maxent version 3.4.4 [34], offering automated functionalities for regularization-multiplier tuning, feature selection, and variable selection based on spatial cross-validation [33]. On a benchmark dataset of over 200 species [42], it was demonstrated that spatialMaxent models outperform those produced using traditional Maxent methods on spatially independent test data [33].

**Fig. 2:**
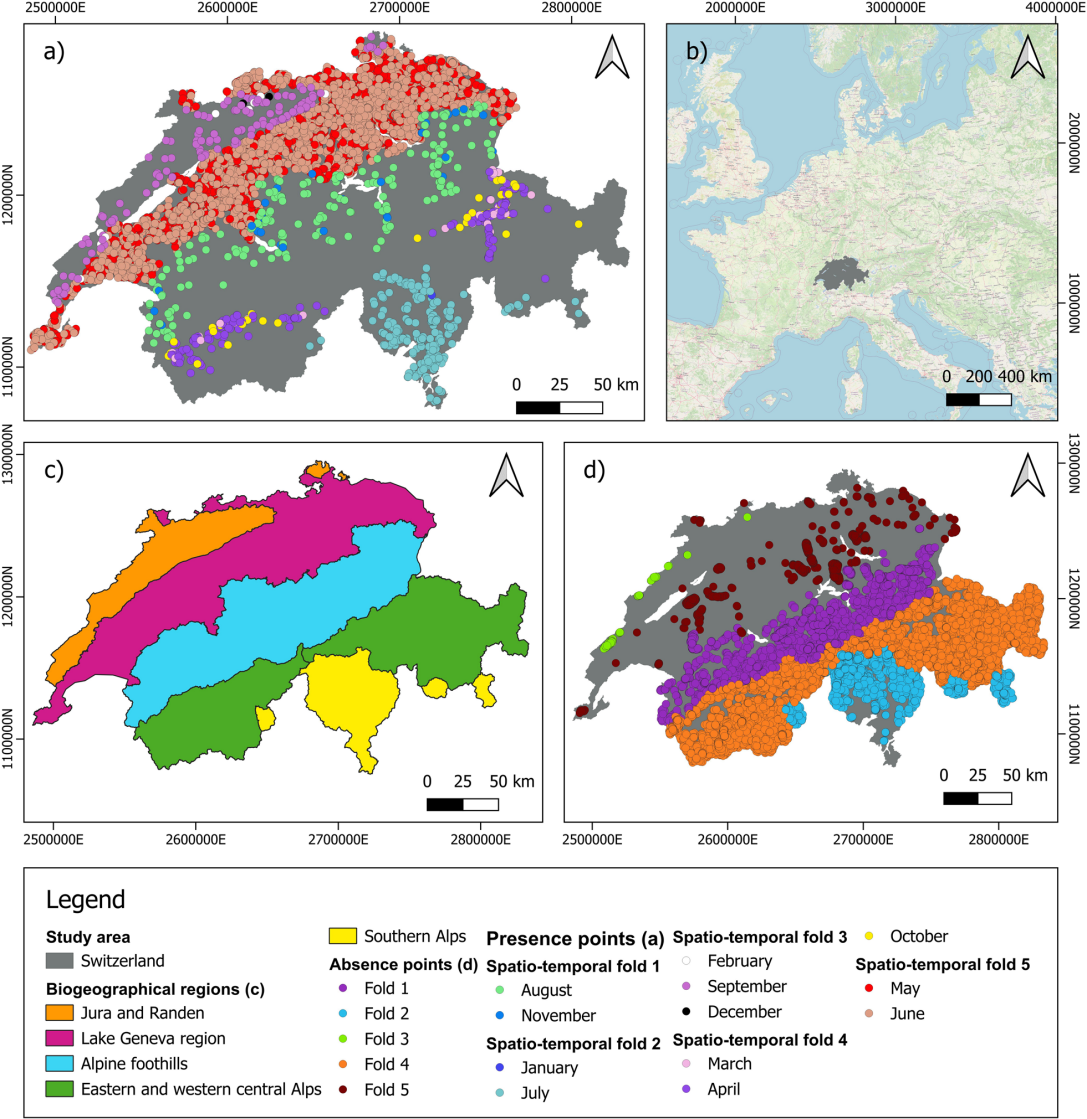
Switzerland: Study area and data records. **a** Records of tick attachment to humans, segregated into spatio-temporal folds based on the biogeographical regions in **c**. Each month is exclusively utilized in one spatio-temporal fold to ensure spatio-temporal independence. **b** The geographical position of Switzerland within Central Europe. **c** Biogeographical regions of Switzerland employed in creating the cross-validation and testing folds. **d** Artificial absence points derived from lakes or regions where the annual mean temperature is below $4^{\circ }\hbox {C}$, separated according to the biogeographical regions in **c**

For the training, validation, and testing of the model with spatio-temporal data, the data were partitioned into five folds using the biogeographical regions of Switzerland (Fig. 2) for spatial separation [43]. For temporal separation, the data were divided by months, ensuring that each month occurred in only one of the five spatial folds during spatial cross-validation (Fig. 2).

With these data, a model was trained with five fold spatio-temporal cross-validation, variable selection, feature selection, and regularization multiplier tuning (for details see [33]). This model was used to create 84 maps of the risk of monthly tick attachment to humans from 2015 to 2021 (Fig. 1; supplementary information).

### Study area

This study focuses on the distribution of tick attachment to humans in Switzerland. Switzerland is centrally located in Europe (Fig. 2b) and is dominated by mountains, primarily the Alps, covering 70% of its territory. However, the majority of the population resides in the relatively flat and hilly “Mittelland” [44]. Overall, the Swiss territory spans 41,285 $\hbox {km}^{2}$ [44], with approximately 8.7 million residents [45]. In many regions of Switzerland, ticks are vectors for multiple pathogens simultaneously and can occur in both rural and urban areas [10, 46]. In addition to transmitting the pathogens for the commonly occurring diseases Lyme borreliosis and TBE, Stegmüller et al. [46] discovered that ticks in Switzerland are also vectors for Alongshan virus.

### Tick reports

**Fig. 3:**
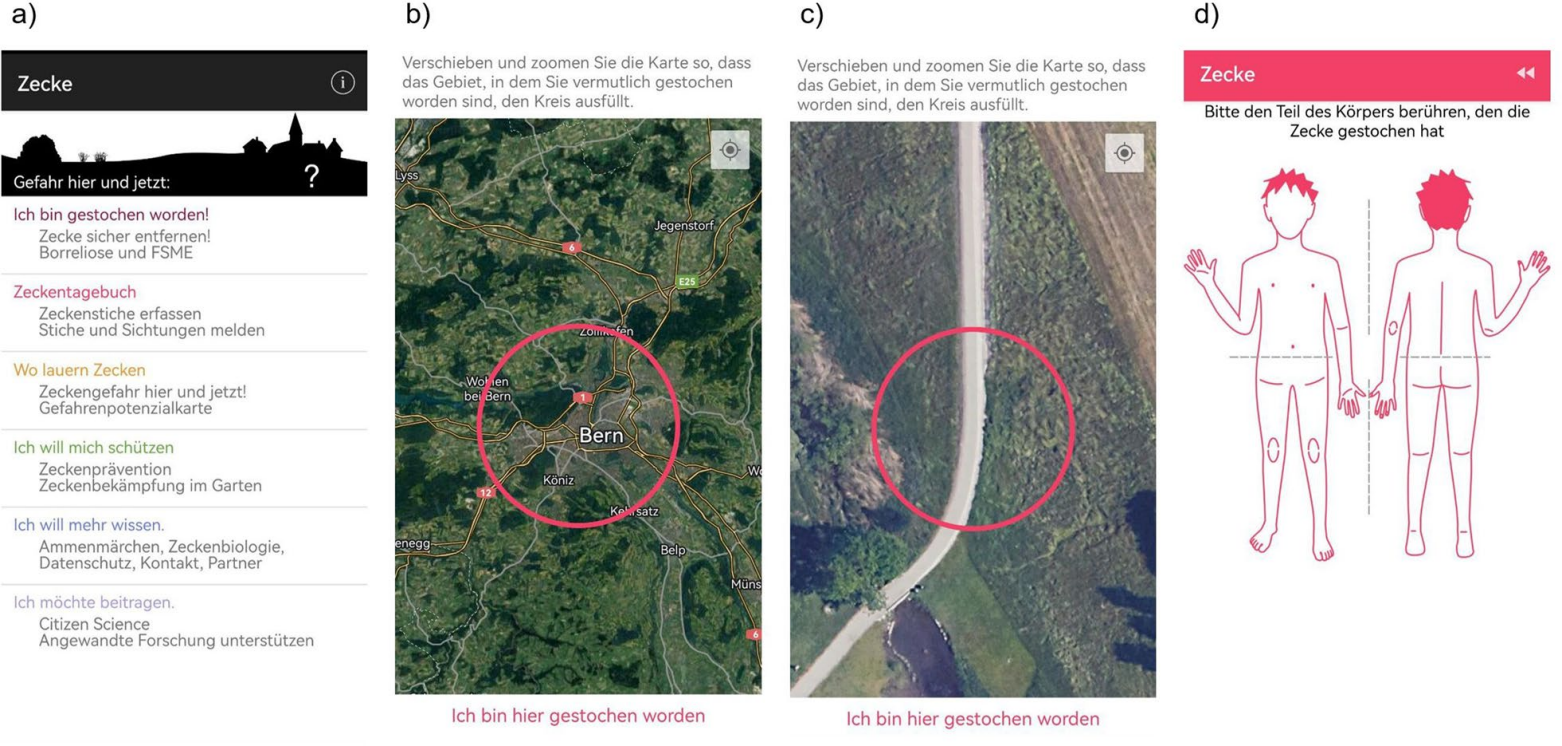
Tick Prevention app. **a** Main menu of the app in German. Here users can find information on how to protect themselves from tick bites, where ticks can be found, and what to do if they have been bitten by a tick. **b**, **c** The setting in which the user can enter where they were bitten by a tick. Users can specify the precision of the location, ranging from very imprecise, e.g., in Bern (**b**), to very precise, e.g., on this specific field path (**c**). **d** The functionality of the app where users can record the specific location on the body where they were bitten

**Fig. 4:**
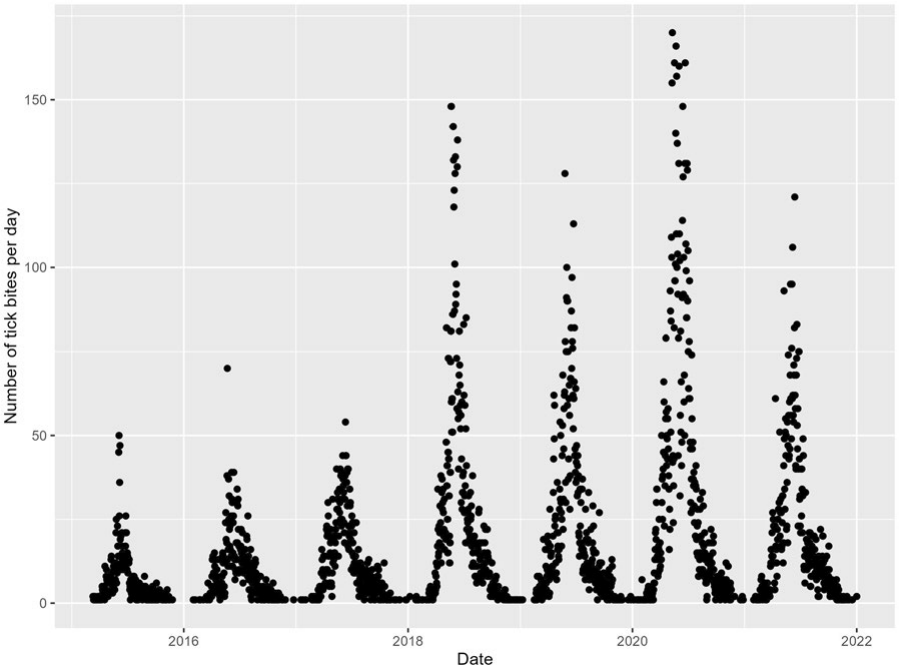
Entries in the Tick Prevention app over time. The *y*-axis indicates the number of entries per day, and the *x*-axis represents the time

The recent availability of field-collected tick data in Switzerland is limited, as the last significant collection effort was conducted by the Swiss Army in 2009 (see [47]). This scarcity of recent field data necessitates alternative approaches to monitor ticks in Switzerland. To obtain good results for mapping tick attachment to humans without conducting additional field campaigns, a citizen science approach was used. The data on tick attachment to humans were collected through the Tick Prevention app developed at the Zurich University of Applied Sciences and operated by the ZHAW spin-off A&K Strategy Ltd. [22]. Users can log both the location and the time of each tick bite (Fig. 3). Since its launch in 2015, this application has accumulated a large dataset (Fig. 4). We only used data where users were confident that the tick bite occurred within a 1 km radius of the reported location, resulting in a dataset of 39,235 tick bites documented between 2015 and 2021. We removed duplicates, specifically records of tick attachments to humans occurring in the same year and month and on the same raster pixel. The tick data were then partitioned into the five biogeographical spatial folds and refined to ensure that each month was represented in only one spatial fold (see Section "Modeling approach"). This processing resulted in a dataset consisting of 10,292 distinct records.

### Background points/pseudo-absences

Maxent is a PO modeling method and therefore requires background points for modeling [34, 36] (see Section "Modeling approach"). The number of background points used for modeling should be sufficiently large to comprehensively represent the entire variable space [37]. While the default setting of Maxent employs 10,000 background points, it has been suggested that this quantity may be insufficient for larger regions [48, 49]. Given that our study encompasses the entire country of Switzerland with a size of 41,285 $\hbox {km}^{2}$ [44], we opted to use more than the usual 10,000 points to ensure a comprehensive representation of the entire variable space. In this study, we modeled 84 monthly time steps from January 2015 to December 2021. For each of these time steps, 1000 background points were randomly sampled over the whole study area using the randomPoints() function from the R package dismo [50], resulting in a total of 84,000 background points.

### Environmental variables

Environmental variables are measurements obtained through methods such as remote sensing, climate monitoring stations, or field studies, and are used to describe and analyze various aspects and conditions of an environment. These variables are shown as grid cells, where each cell contains a value for a specific environmental characteristic. In the context of modeling tick attachment to humans, a comprehensive set of environmental variables was employed, covering variables produced for local (Switzerland), regional (Europe), and global scales. In modeling tick attachment to humans, environmental variables were selected based on their possible impact on tick occurrence, and human activity. Garcia-Martí et al. [51] described in their study the importance of weather data, vegetation data from satellites, and land-cover data for mapping tick dynamics. Building on this approach, we extended the set of environmental variables by incorporating variables that reflect human behavior in space, such as population density. The variables were acquired across diverse temporal scales, including monthly, yearly, seasonal (distinct datasets for spring, summer, autumn, and winter), and static variables. The variables used in this study (Table 1) fall into one of the following categories: land-cover data, population data, weather data, vegetation indices, terrain data, and roe deer data.

Vegetation indices can be useful when mapping ticks [51]. Therefore, we used three spectral vegetation indices to capture spectral vegetation properties: the enhanced vegetation index (EVI) [52], which ranges from $-1$ (bare soil) to +1 (dense vegetation), the leaf area index (LAI) [53], which measures leaf area per ground area, and the green chlorophyll index (GCI) [54], which estimates chlorophyll content in vegetation.

Furthermore, land cover influences the likelihood of tick–host encounters (e.g., with deer, humans, or mice) and can thus serve as a critical factor affecting tick occurrence [51]. Consequently, we incorporated multiple land-cover datasets, including CORINE (Coordination of Information on the Environment) land-cover data, Swiss land-cover data, Swiss forest composition, global forest cover fraction, and global cropland distribution (see Table 1).

The population data used in this study include Swiss population data, worldwide population data, human footprint data, and global travel time to cities. These datasets were selected to capture human presence and activity, which could influence tick attachment to humans. The population data provide population densities, the human footprint data reflect areas of high human activity, and global travel time to cities reflects accessibility, which affects human movement in space.

Weather data were also incorporated, as weather conditions can influence tick behavior by determining the onset of the questing season or affecting survival during winter [51]. The weather data used in this study include annual snow cover, monthly precipitation, monthly relative sunshine duration, and monthly mean temperature (see Table 1).

We also included terrain data, specifically a digital height model, which provides information on terrain height for all of Switzerland. The roe deer data [55] provide the distribution of roe deer across Europe and were incorporated due to the deer’s role as a host for ticks [56].

To ensure consistency, all environmental variables were resampled to a uniform spatial resolution of 100 m. Further details on the native resolution, data sources, their temporal resolution, and other relevant information for the environmental variables are provided in Table 1.

### Absence points

A variety of metrics are available to assess the performance of a species distribution model. However, many of these metrics, such as the area under the receiver operating characteristic curve ($\hbox {AUC}_{\textrm{ROC}}$ [57]), cannot be calculated on the presence points of a species alone. In addition to the presence points, information indicating the absence of a species is needed. Absence points were generated artificially in this study for model testing. It is known that ticks do not survive in areas where the average annual temperature is below $4^{\circ }\hbox {C}$; therefore, the mean monthly temperature variables were averaged to yearly data. From these data, areas warmer than $4^{\circ }\hbox {C}$ were masked, and 10,000 absence points were randomly sampled for each year using the randomPoints() function from the R package dismo [50]. Furthermore, ticks cannot survive in water; therefore, on the lakes in the study area, 30,000 absence points were sampled using the same function. These absence points were used solely for calculating evaluation metrics (see Section "Evaluation" ), as spatialMaxent effectively handles PO data. By modeling on PO data, we developed a model solely on citizen science data, which may be of interest for similar citizen science projects. This approach also allowed tick experts in Switzerland to conduct an initial assessment of the model without introducing the uncertainty associated with artificial absence points.

### Evaluation

For evaluating the performance of a species distribution model, Konowalik and Nosol [58] advised incorporating expert opinion along with multiple performance metrics. Two experts for ticks in Switzerland were therefore consulted to assess the maps. Furthermore, several evaluation metrics were calculated using forward-fold-metric estimation (FFME) [33], which is a form of nested cross-validation [59]. In FFME, not one but two test folds, spatially and temporally independent of each other, were separated from the training data as test data. A model was trained using the remaining training data and evaluated using the two folds removed from the training data for model testing. This procedure was repeated for all possible combinations of two test folds, using 10 different combinations in this study.

Since individual metrics often come with uncertainties [58, 60], a variety of metrics were utilized to obtain a comprehensive assessment of the model’s performance. For metrics requiring absence data, the same number of artificial absence points (Fig. 2c) as presence points in the test data were sampled. The metrics $\hbox {AUC}_{\textrm{ROC}}$, true skill statistic (TSS) [61], and percent correctly classified (PCC) were calculated using the evalSDM() function from the R package mecofun (version 0.1.1) [62]. Additionally, the area under the precision-recall-gain curve ($\hbox {AUC}_{\textrm{PRG}}$) [63] was calculated using the R package prg (version 0.5.1) [64], and the continuous Boyce index (CBI) [65] using the R package ecospat (version 3.5) [66]. The metrics mean absolute error (MAE), root mean square error (RMSE), and mean log loss (logloss) were calculated using the R package Metrics (version 0.1.4) [67]. Furthermore, a Pearson correlation (COR) between observed test data and mapped values was computed using the base R function cor().

**Fig. 5:**
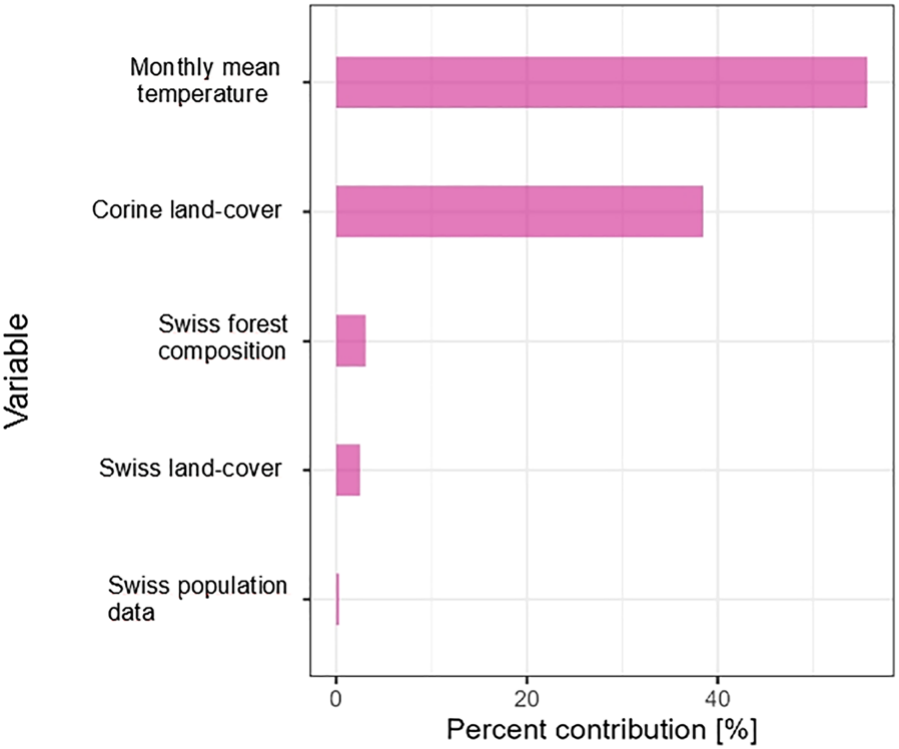
Variable importance of the selected variables. The barplot displays the variables selected as important through the automatic variable selection procedure. The *y*-axis shows the variables, while the *x*-axis indicates the percentage contribution of each variable to the model

**Fig. 6:**
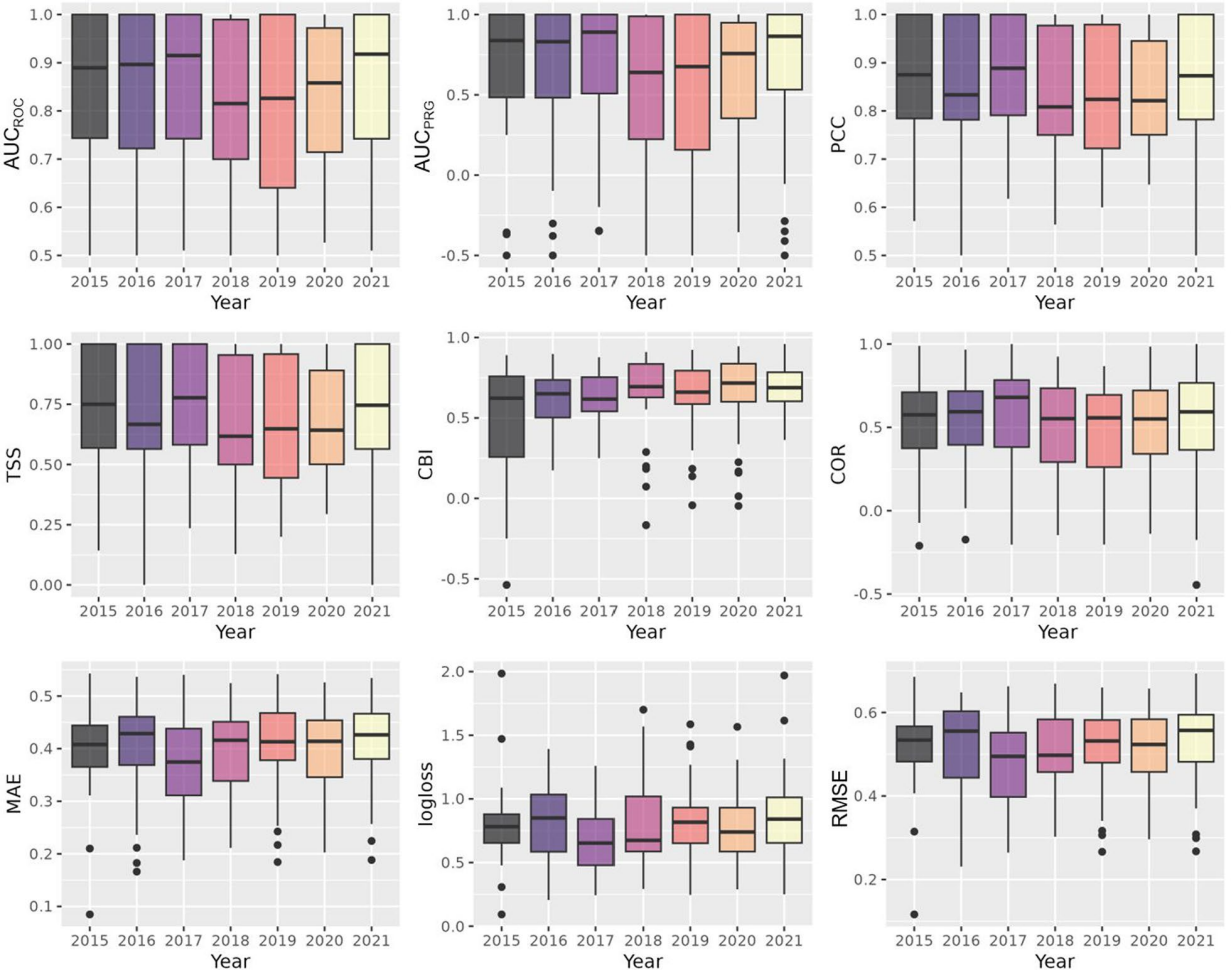
Test results by year. The boxplots depict the test results on the test folds, stratified by year, across nine evaluation metrics: area under the receiver operating characteristic curve ($\hbox {AUC}_{\textrm{ROC}}$), area under the precision-recall-gain curve ($\hbox {AUC}_{\textrm{PRG}}$), percent correctly classified (PCC), true skill statistic (TSS), continuous Boyce index (CBI), Pearson correlation (COR), mean absolute error (MAE), root mean square error (RMSE), and mean log loss (logloss). The *x*-axis represents the year, while the *y*-axis indicates the metric

**Fig. 7:**
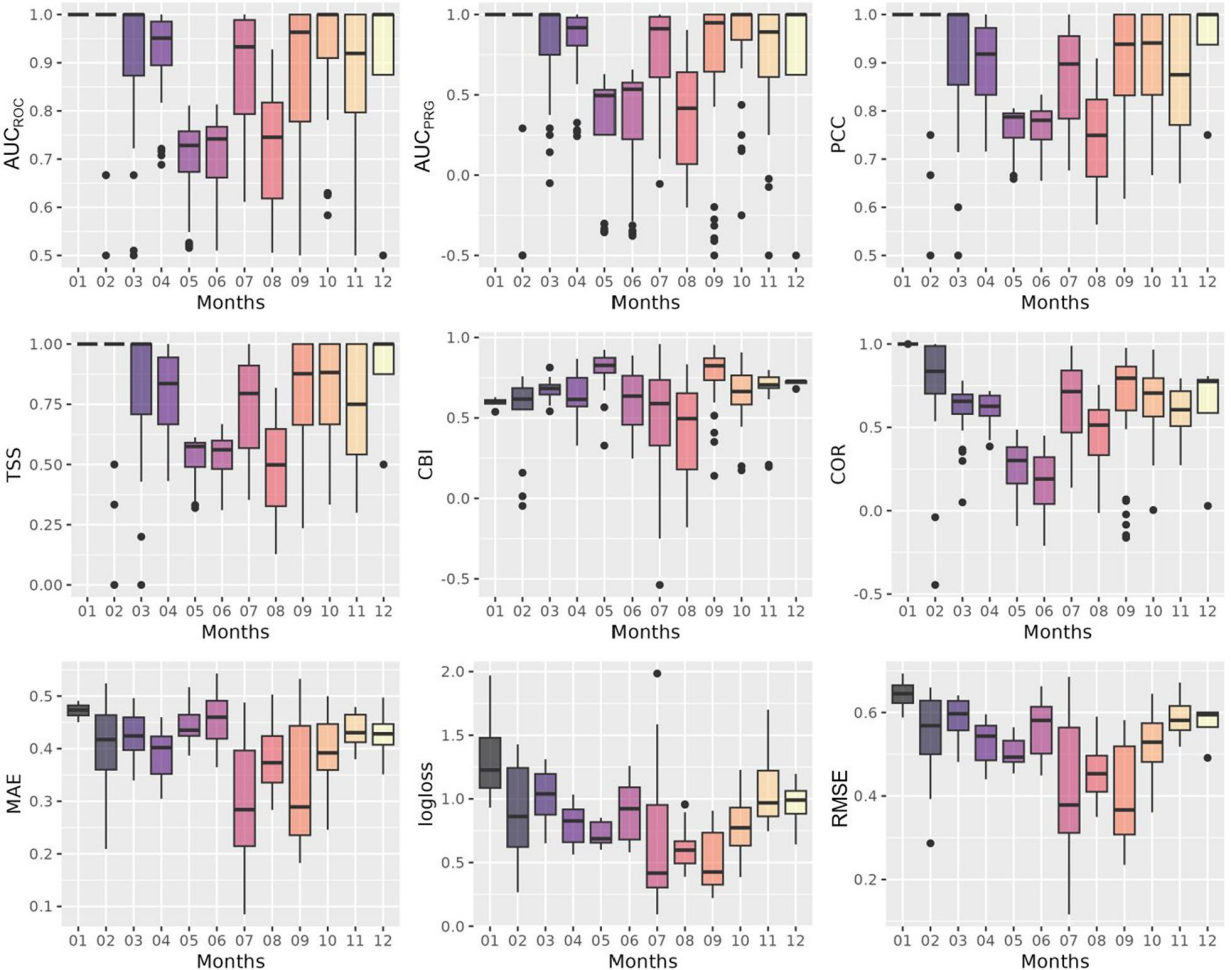
Test results by month. The boxplots depict the results on test folds, stratified by month, across nine evaluation metrics: area under the receiver operating characteristic curve ($\hbox {AUC}_{\textrm{ROC}}$), area under the precision-recall-gain curve ($\hbox {AUC}_{\textrm{PRG}}$), percent correctly classified (PCC), true skill statistic (TSS), continuous Boyce index (CBI), Pearson correlation (COR), mean absolute error (MAE), root mean square error (RMSE), and mean log loss (logloss). The *x*-axis represents the month, while the *y*-axis indicates the evaluation metric

**Fig. 8:**
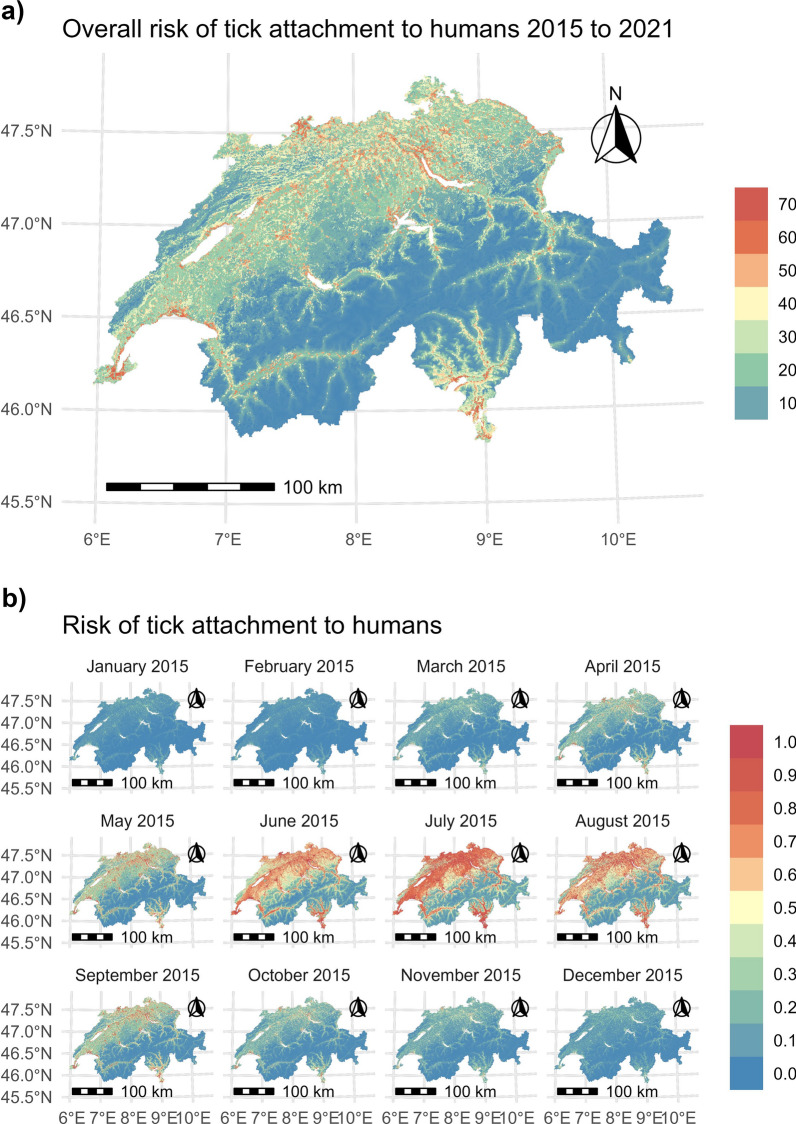
Maps depicting the risk of tick attachment to humans in Switzerland. **a** The overall risk of tick attachment to humans across Switzerland, derived from the sum of all 84 monthly maps from 2015 to 2021. The scale ranges from 0 (indicating low overall risk for the entire period) to 70 (indicating high overall risk for the entire period). **b** The monthly risk of tick attachment for the year 2015, selected as an example. The complete set of monthly maps for all years is available as time series in the supplementary information. The scale ranges from 0 (low risk) to 1 (high risk)

**Fig. 9:**
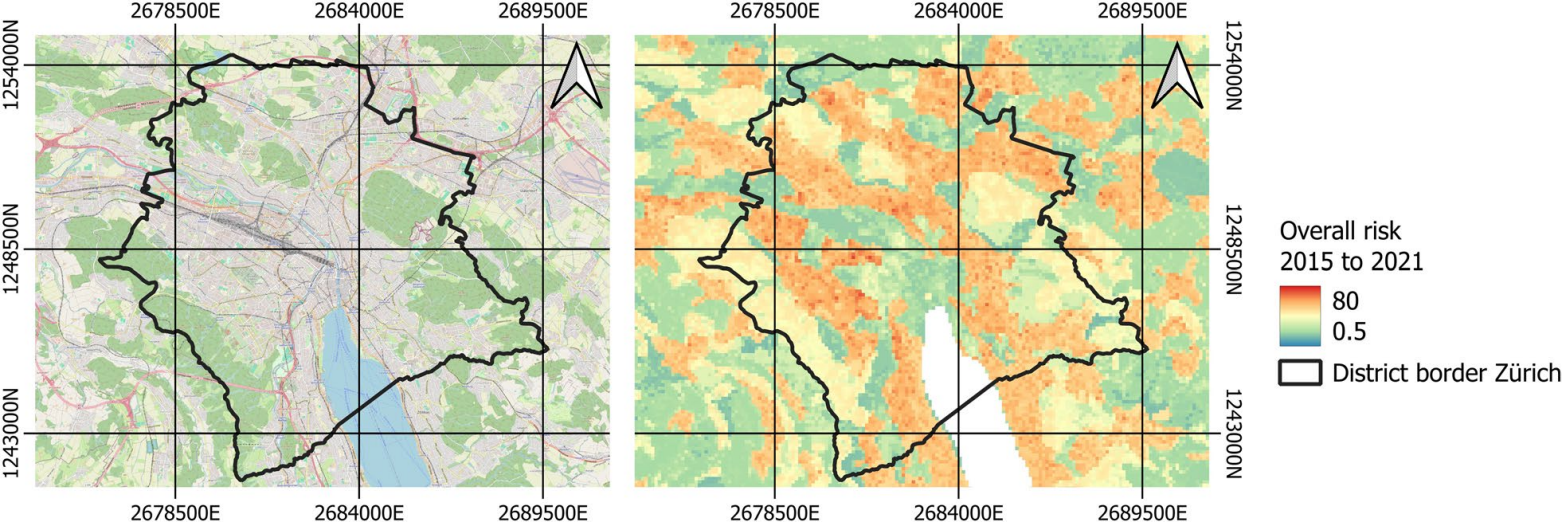
The overall risk of tick attachment to humans in Zürich. On the left, an OpenStreetMap map of the Zürich region is shown. On the right, the overall risk of tick attachment to humans is depicted, with yellow and red colors indicating high overall risk, while green colors represent low risk. Data: Federal Office of Topography Swisstopo [84]

**Table 1:** Overview of environmental variables used in the study and their spatial and temporal resolution

Variable name	Native spatial resolution	Temporal resolution	Data source/reference	Additional description
Digital height model	25 m	Static	Swisstopo; https://www.swisstopo.admin.ch/en/height-model-dhm25	
Annual snow cover	1 km	Annual	EOC Geoservice; https://geoservice.dlr.de/web/	
Swiss population data	NA	Annual	Swiss Federal Statistical Office (Bundesamt für Statistik der Schweiz; https://www.bfs.admin.ch)	Data on population count and density. Values in areas where no people reside were assigned a value of 0
Enhanced vegetation index (EVI)	30 m	Seasonal and annual	Swiss Data Cube https://www.swissdatacube.org/; 2010–2019: https://doi.org/10/gqw3gn; 2020–2021: https://doi.org/10/gqwwtk	Analysis-ready earth observation data for Switzerland. Seasonal median (spring, summer, autumn, and winter) or annual data
Leaf area index (LAI; Boegh et al. [53])	30 m	Seasonal and annual	Swiss Data Cube https://www.swissdatacube.org/; 2010–2019: https://doi.org/10/gqw3gj; 2020–2021: https://doi.org/10/gqwwtg	Analysis-ready earth observation data for Switzerland. Seasonal median (spring, summer, autumn, and winter) or annual data
Green chlorophyll index (GCI; Gitelson et al. [54])	30 m	Seasonal and annual	Swiss Data Cube https://www.swissdatacube.org/; 2010–2019: https://doi.org/10/gqxdkd; 2020–2021: https://doi.org/10/gqwwtf	Analysis-ready earth observation data for Switzerland. Seasonal median (spring, summer, autumn, and winter) or annual data
Worldwide population data	1 km	Annual	CIESIN [85]; https://doi.org/10.7927/H49C6VHW	Gridded population of the world
Human footprint	1 km	Annual	Mu et al. [86]; https://doi.org/10.6084/m9.figshare.16571064	Annual terrestrial human footprint data
Global travel time to cities	1 km	2015	Nelson et al. [87]; https://doi.org/10.6084/m9.figshare.7638134.v3	Global accessibility indicators for travel time to cities. Pixel values indicating travel time in minutes from each pixel to the nearest settlement across different settlement classes (e.g., $\ge$5000 and <10,000 people)
Monthly precipitation (RhiresM)	1 km	Monthly	MeteoSwiss; https://www.meteoschweiz.admin.ch; data are not publicly accessible	
Monthly relative sunshine duration (SrelM)	1 km	Monthly	MeteoSwiss; https://www.meteoschweiz.admin.ch; data are not publicly accessible	
Monthly mean temperature (TabsM)	1 km	Monthly	MeteoSwiss; https://www.meteoschweiz.admin.ch; data are not publicly accessible	
CORINE land-cover	NA	2018	European Environment Agency (EEA), 2018 [88]	44 Distinct land-cover classes for Europe as polygon data
Swiss land-cover	NA	2018	Swiss Federal Statistics Office; www.bfs.admin.ch	Contains 72 land-cover classes
Swiss forest composition	10 m	2018	Swiss federal authorities; http://data.geo.admin.ch/ch.bafu.landesforstinventar-waldmischungsgrad/Waldmischungsgrad_2018_10m_2056.tif	Proportion of deciduous trees within the forested areas
Global forest fraction	1 km	Annual	Winkler et al. [89]; https://doi.org/10.1594/PANGAEA.921846	
Global cropland data	1 km	Annual	Cao et al. [90]	Proportion of cropland by year
Roe deer data	1 km	2014	Alexander et al. [55]; https://doi.org/10.6084/m9.figshare.1008335.v1	Distribution of roe deer in Europe

These variables are input into the variable selection algorithm but are not necessarily all part of the final model (see Section "Modeling approach"). Globally available datasets were accessed via download links provided at https://github.com/OpenGeoHub/spatial-prediction-eml/blob/master/input/mood_layers1km.csv

## Results

### Selected model parameters

Model tuning revealed that the optimal model parameters were a beta multiplier of 6.5 and the use of the linear feature transformation. Five important variables were selected during the variable selection process: monthly mean temperature, CORINE land-cover class, Swiss forest composition, Swiss land-cover, and Swiss population data (Fig. 5). The most important variables were monthly mean temperature and CORINE land-cover, which contributed 55.7% and 38.5% to the model, respectively. The remaining variables initially considered (Table 1) were excluded from the final model, as they did not enhance model performance based on the algorithmic variable selection process (see Section "Modeling approach"). This exclusion ensured that only variables with high performance were retained, preventing overfitting of the model.

### Assessment of model performance

To assess the model performance on the test data, the metrics were analyzed separately by years and months. The differences in the metrics between individual years (Fig. 6) were not as high as those between the different months (Fig. 7). However, the metrics $\hbox {AUC}_{\textrm{ROC}}$, $\hbox {AUC}_{\textrm{PRG}}$, PCC, and TSS performed slightly lower for the years 2018 to 2020 (Fig. 6; Appendix A), while the results for the year 2017 were somewhat higher than for other years, with the exception of CBI (0.62; Appendix A). The second-best results were achieved for the year 2021, which attained the second-best scores for the metrics $\hbox {AUC}_{\textrm{ROC}}$ (0.92), TSS (0.75), $\hbox {AUC}_{\textrm{PRG}}$ (0.86), COR (0.59), and CBI (0.69; Fig. 6; Appendix A).

When analyzing the results over multiple months, the variability between individual months was greater than the variability between years (Fig. 7). In particular, the winter months showed good results, during which low tick activity can be expected (January, February, and December). These months achieved the highest score of 1 for the metrics $\hbox {AUC}_{\textrm{ROC}}$, TSS, PCC, and $\hbox {AUC}_{\textrm{PRG}}$ (Fig. 7; Appendix A). Good results were also achieved for COR (January 1, February 0.84, December 0.78; Appendix A), while poorer values were obtained for the metrics MAE, RMSE, and logloss. Some of the results contrasted with each other. For example, March had very good values for the metrics $\hbox {AUC}_{\textrm{ROC}}$ (1), TSS (1), PCC (1), $\hbox {AUC}_{\textrm{PRG}}$ (1), COR (0.66), and CBI (0.68), while it ranked in the middle for MAE (0.42) and obtained one of the worst ranks for RMSE (0.6) and logloss (1.04) compared to the other months (Fig. 7; Appendix A). In comparison to the other months, May, June, and August exhibited the weakest performance. The metrics $\hbox {AUC}_{\textrm{ROC}}$, TSS, $\hbox {AUC}_{\textrm{PRG}}$, and COR had the lowest overall performance values during these months (Fig. 7; Appendix A).

### Time series maps and overall risk

A total of 84 maps depicting the risk of tick attachment to humans in Switzerland were generated in this study. All maps from 2015 to 2021 are accessible as a time series in the supplementary information, while the maps for 2015 are shown as examples in Fig. 8b.

The risk of tick attachment to humans for most years from 2015 to 2021 increased from April onwards, expanding to more regions and peaking in July (see time series in supplementary information). During this peak, a large part of the populated Switzerland experienced high risk, which then declined, with only a few regions maintaining high risk by September. Analysis of the overall risk over the entire period from January 2015 to December 2021, based on the summed risk values, reveals that tick attachment to humans is particularly high at the edges of settlement areas (Fig. 8a). In particular, tick attachment rates are notably higher in sparsely built-up suburban areas with green spaces, whereas they are lower in urban areas. Additionally, forested areas adjacent to cities also exhibit heightened risk levels (Fig. 9).

## Discussion

In this study, the risk of tick attachment to humans in Switzerland was mapped at a spatial resolution of 100 m on a monthly basis from 2015 to 2021. A comprehensive dataset collected by a citizen science approach through the Tick Prevention app of Switzerland was utilized to create the maps.

The time series of monthly maps revealed higher risk in suburban areas with green spaces and adjacent forested regions, consistent with previous findings, suggesting that urban and suburban areas can harbor high tick populations [68–70]. Furthermore, Oechslin et al. [10] demonstrated that ticks found in urban and suburban regions of Switzerland exhibit carrier rates of tick-borne diseases comparable to those in rural regions. This suggests that the urban and suburban areas identified as high risk for tick attachment to humans in our study may warrant increased attention for public health management.

The maps for the months of May and June display lower metric scores compared to other months across all years (Fig. 7). Given that tick activity typically begins in early spring [71], the low risk depicted in these months suggests a potential underestimation of the risk. For example, compared to July of most years, we observe lower risk levels during these two months (e.g., as seen for 2015 in Fig. 8b). However, an examination of Lyme disease cases on the Infectious Diseases Dashboard of the Swiss Federal Office of Public Health (FOPH; https://www.idd.bag.admin.ch/diseases/lyme/statistic; count for the years 2015 to 2021; accessed on 30.08.2024) reveals that there is no consistent pattern regarding which month has the highest number of reported cases in Switzerland. In 2021 and 2022, the highest number of cases was reported in June, whereas in 2015, 2020, and 2023, the peak occurred in July. This indicates a shift from the typical pattern of two distinct peaks in June and September, observed in previous decades, to a more diffuse pattern. Considering these changes, the lower metric values observed in May and June could be due to shifting tick activity patterns or complexities that the model may struggle to capture, warranting further investigation to improve predictive accuracy. Additionally, it is important to note that the results presented here are specific to the modeling strategy used in this study, as the variable selection, validation strategy, and modeling method can have a substantial impact on the model outcomes [37, 38, 72].

Furthermore, the citizen science approach, while valuable for collecting such a large dataset on tick attachment to humans, also has its limitations. The quality of the data relies heavily on the users of the app [21], and more detailed information, such as the identification of tick species or the potential transmission of tick-borne diseases, could not be acquired. Moreover, it does not include absence points, which led us to create artificial absence points. This approach may limit the reliability of the calculated evaluation metrics and, consequently, the model’s assessment. For example, the use of temperature thresholds to create artificial absence points may overlook microclimatic variations where ticks can survive, potentially affecting the accuracy of the evaluation metrics. However, after discussions with the tick experts on our author team, we opted for this simple yet pragmatic approach, acknowledging that temperature serves as a suitable proxy for distribution limitations in the predominantly terrain-dominated landscape of Switzerland.

Our study highlighted the high importance of the variables monthly mean temperature, CORINE land-cover , Swiss forest composition, Swiss land-cover, and Swiss population data for modeling tick attachment to humans. This indicates a clear preference of the model for regional datasets over more generalized, globally available ones. The selected variables align with many variables mentioned in the literature that are pertinent to tick occurrence; for example, the dependence on temperature is mentioned frequently [71, 73–75]. The preference of ticks for specific forest types has also been observed [76–78]. The importance of the land-cover variables is probably not only due to tick distribution but also if areas are frequently visited by humans, such as for recreational purposes [79]. For example, Salkeld et al. [16] investigated human exposure to ticks and found that outdoor recreation significantly affects human exposure in the USA. The variable monthly mean temperature is the most important dynamic variable in the model; therefore, the changes across the maps (see supplementary information) are driven largely by weather conditions. While this captures a seasonal impact of tick attachment to humans, the static nature of the other variables limits the model’s ability to reflect temporal changes, such as shifts in land use; these limitations should be considered when interpreting the maps. In this regard, a fine-grained time series of land-cover changes could be beneficial.

Rochat et al. [75] mapped the distribution of *Ixodes ricinus* in Switzerland for June 2009 and June 2018. The spatial distribution of *I. ricinus* in their June 2018 map closely aligns with the risk of tick attachment to humans that we mapped for the same month. Although this congruence suggests a linkage between tick habitat suitability and the observed tick attachment risk, we would emphasize here that we modeled tick attachment to humans and not the habitat suitability for ticks. However, this connection suggests a correlation between tick habitat suitability and tick attachment to humans which was also observed by Ribeiro et al. [78].

Following this study, it would be beneficial to predict the risk of tick attachment to humans in Switzerland using future climate data. Such predictions could provide timely warnings to the population, potentially several months before the actual risk arises, allowing for preventive public health responses. Additionally, long-term predictions, incorporating diverse climate change scenarios (e.g., using CHELSEA data [80]) could enable more robust and extended planning for the healthcare system. It could also be beneficial to extend the use of the Tick Prevention app to other countries facing similar challenges. Furthermore, the app could be enhanced to collect more detailed data by enabling users to submit tick images for species classification [81, 82] or by facilitating the submission of ticks to research laboratories for in-depth analysis [83].

Our study offers insights into the spatial and temporal dynamics of tick attachment to humans in Switzerland by leveraging citizen science data alongside a state-of-the-art modeling approach. While the results must be interpreted with caution due to the uncertainties of the citizen science data and the potential limitations of evaluation metrics calculated with artificial absence points, as well as the influence of modeling strategies on the outcomes, these maps represent the first high-resolution depiction of tick attachment to humans in Switzerland. They can serve as a foundation for future research aimed at informing targeted interventions and public health strategies to reduce the incidence of tick-borne illnesses in the country. Our work also highlights the potential value of citizen science in epidemiological surveillance. To translate these insights into actionable outcomes, it is crucial to further strengthen collaboration among public health authorities, researchers, and the public.

## Supplementary Information

Additional file 1.

## Data Availability

All code used for data processing and to create the models used for mapping tick attachment to humans is publicly available at: https://github.com/envima/TickAttachmentSwitzerland2024.

## Appendix A

See Tables 2 and 3.

**Table 2:** Summary of model testing results aggregated by year

Year	$\hbox {AUC}_{\textrm{ROC}}$	$\hbox {AUC}_{\textrm{PRG}}$	PCC	TSS	CBI	COR	MAE	logloss	RMSE
2015	0.89	0.84	0.88	0.75	0.62	0.58	0.41	0.78	0.53
2016	0.9	0.83	0.83	0.67	0.65	0.59	0.43	0.85	0.56
2017	0.92	0.89	0.89	0.78	0.62	0.68	0.37	0.65	0.49
2018	0.82	0.64	0.81	0.62	0.69	0.55	0.42	0.67	0.5
2019	0.83	0.68	0.82	0.65	0.66	0.56	0.41	0.82	0.53
2020	0.86	0.76	0.82	0.64	0.72	0.55	0.41	0.74	0.52
2021	0.92	0.86	0.87	0.75	0.69	0.59	0.43	0.84	0.56

The table displays median results from model testing across various test folds (see Section "Evaluation") for the years 2015 to 2021. The analysis included the following metrics: area under the receiver operating characteristic curve ($\hbox {AUC}_{\textrm{ROC}}$), area under the precision-recall-gain curve ($\hbox {AUC}_{\textrm{PRG}}$), percent correctly classified (PCC), true skill statistic (TSS), continuous Boyce index (CBI), Pearson correlation (COR), mean absolute error (MAE), root mean square error (RMSE), and log loss (logloss). Values rounded to two decimal digits

**Table 3:** Summary of model testing results aggregated by month

Month	$\hbox {AUC}_{\textrm{ROC}}$	$\hbox {AUC}_{\textrm{PRG}}$	PCC	TSS	CBI	COR	MAE	logloss	RMSE
1	1	1	1	1	0.6	1	0.47	1.23	0.65
2	1	1	1	1	0.62	0.84	0.42	0.86	0.57
3	1	1	1	1	0.68	0.66	0.42	1.04	0.6
4	0.95	0.92	0.92	0.84	0.62	0.63	0.4	0.83	0.54
5	0.73	0.5	0.79	0.57	0.83	0.3	0.44	0.69	0.49
6	0.74	0.53	0.78	0.56	0.64	0.19	0.46	0.92	0.58
7	0.93	0.91	0.9	0.79	0.59	0.71	0.28	0.42	0.38
8	0.75	0.42	0.75	0.5	0.5	0.51	0.37	0.6	0.45
9	0.96	0.95	0.94	0.88	0.82	0.8	0.29	0.43	0.37
10	1	1	0.94	0.88	0.66	0.71	0.39	0.77	0.53
11	0.92	0.89	0.88	0.75	0.7	0.61	0.43	0.97	0.58
12	1	1	1	1	0.73	0.78	0.43	0.99	0.59

The table displays median results from model testing across various test folds (see Section "Evaluation") for all 12 months. The analysis included the following metrics: area under the receiver operating characteristic curve ($\hbox {AUC}_{\textrm{ROC}}$), area under the precision-recall-gain curve ($\hbox {AUC}_{\textrm{PRG}}$), percent correctly classified (PCC), true skill statistic (TSS), continuous Boyce index (CBI), Pearson correlation (COR), mean absolute error (MAE), root mean square error (RMSE), and log loss (logloss). Values rounded to two decimal digits

## Abbreviations

TBE: Tick-borne encephalitis
SDM: Species distribution modeling
PA: Presence–absence
PO: Presence-only
EVI: Enhanced vegetation index
$\hbox {AUC}_{\textrm{ROC}}$: Area under the receiver operating characteristic curve
FFME: Forward-fold-metric-estimation
TSS: True skill statistic
PCC: Percent correctly classified
$\hbox {AUC}_{\textrm{PRG}}$: Area under the precision-recall-gain curve
CBI: Continuous Boyce index
MAE: Mean absolute error
RMSE: Root mean square error
COR: Pearson correlation
FOPH: Swiss Federal Office of Public Health

## Footnotes

1 https://www.hopkinslymetracker.org/overview/, accessed 22.06.2024.

2 https://www.cdc.gov/lyme/, accessed 22.06.2024
